# Seropositivity against rubella virus envelope protein 1, but not 2, is associated with an increased risk of multiple sclerosis

**DOI:** 10.1002/cti2.70094

**Published:** 2026-04-16

**Authors:** Jens Ingvarsson, Viktor Grut, Martin Biström, Linn Persson Berg, Julia Butt, Birgitta Michels, Pernilla Stridh, Tomas Olsson, Lars Alfredsson, Ingrid Kockum, Tim Waterboer, Staffan Nilsson, Peter Sundström

**Affiliations:** ^1^ Department of Clinical Sciences, Neurosciences Umeå University Umeå Sweden; ^2^ Department of Infectious Diseases, Institute of Biomedicine University of Gothenburg Gothenburg Sweden; ^3^ Department of Clinical Microbiology Sahlgrenska University Hospital Gothenburg Sweden; ^4^ Division of Infections and Cancer Epidemiology German Cancer Research Center Heidelberg Germany; ^5^ Department of Clinical Neuroscience Karolinska Institutet Stockholm Sweden; ^6^ Center for Molecular Medicine Karolinska University Hospital Stockholm Sweden; ^7^ Institute of Environmental Medicine Karolinska Institutet Stockholm Sweden; ^8^ Department of Laboratory Medicine, Institute of Biomedicine University of Gothenburg Gothenburg Sweden

**Keywords:** multiple sclerosis, neurofilament light‐chain, rubella virus, serology, vaccination

## Abstract

**Objective:**

Several factors have been associated with altered risk of developing multiple sclerosis (MS). Epstein–Barr virus (EBV) is the critical risk factor for MS, but recent studies indicate that human herpesvirus 6A (HHV‐6A) and cytomegalovirus (CMV) are involved in MS aetiopathogenesis. We recently reported an association between rubella virus (RV) envelope protein 1 (E1) seropositivity and the risk of developing MS. Here, we aimed to further explore this association.

**Methods:**

Bio‐banked blood samples collected before the onset of MS from 981 cases and 1278 matched controls were analysed for antibodies against RV envelope proteins 1 and 2 (E2), and serum‐neurofilament light chain (S‐NfL), a marker for axonal injury. The association between RV and MS was analysed with conditional logistic regression, calculating odds ratios (OR) with 95% confidence intervals (CI).

**Results:**

Rubella E1 seropositivity was significantly associated with a higher risk of MS development, even when adjusting for EBV, CMV and HHV‐6A serostatus (OR = 1.67, 95% CI 1.29–2.14, *P* < 0.001). We also observed an increase in seroreactivity against RV E1 before an increase in S‐NfL in MS cases, indicating that increased RV E1 seroreactivity precedes axonal injury in the MS prodrome at group‐level. There was no association between RV E2 serostatus and MS risk. The association between RV E1 serostatus and MS risk remained similar when the analysis was restricted to unvaccinated participants (OR = 1.78, 95% CI 1.07–2.98, *P* = 0.028).

**Conclusions:**

Our results support a broadening of the viral hypothesis in MS, arguing for further studies on the neurotropic RV in MS aetiology.

## Graphical abstract

In samples collected before the onset of multiple sclerosis (MS), we show that seropositivity against rubella virus (RV) envelope protein 1 (E1) was associated with increased risk of developing MS. Furthermore, at group level, cases had higher seroreactivity against RV E1 compared to matched controls before a significant increase in neurofilament light chain, a biomarker for neuronal damage. This may suggest an altered immune response against RV E1 before the MS prodrome in people who later develop MS.

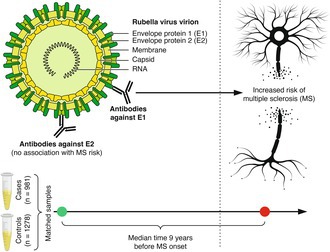

## Background

Multiple sclerosis (MS) is a debilitating immune mediated disease of the central nervous system (CNS), characterised by multifocal demyelination and chronic inflammation.^1^, ^2^ The aetiology of MS remains elusive, despite Epstein–Barr Virus (EBV) having been revealed as a central factor.^3^, ^4^ Several other environmental and genetic factors have been associated with altered risk of developing MS, suggesting a multifactorial aetiology. Such factors include human leukocyte antigen (HLA) variants HLA‐DRB1*15 and HLA‐A*02,^5^, ^6^ previous infection with human herpesvirus 6A (HHV‐6A)^3^, ^7^ or cytomegalovirus (CMV),^8^ smoking^9^ and vitamin D levels.^10^ Recently, we also reported an association between seropositivity for rubella virus (RV) envelope protein 1 (E1) and increased risk of developing MS, in samples drawn before the onset of MS.^11^ These findings have been supported by another recent study based on samples drawn after MS diagnosis.^12^

Viruses have long been regarded as potential triggers of immune‐mediated disease in general, and in MS specifically. Suggested mechanisms include overactive immune response against infected cell types and molecular mimicry, where viral epitopes similar to human protein structures trigger a response against endogenic structures.^13^ To exemplify, molecular mimicry between Epstein–Barr nuclear antigen 1 (EBNA‐1) and anoctamin 2, a chloride channel, has been suggested as a contributing factor in MS pathophysiology.^14^ Regarding RV, the only known receptor for RV is myelin oligodendrocyte glycoprotein (MOG), strongly suggesting that RV can infect oligodendrocytes, which could potentially cause demyelination.^15^ This binding is facilitated by E1, but the virus envelope also contains a second protein, envelope protein 2 (E2), mostly hidden within the virion *in vivo*.^16^ Still, E2 contains at least one region where neutralising antibodies can bind, specifically in the first 115 amino acids (AA) of the protein.^17^ To our knowledge, there is only one previous study comparing serological response to RV E1 and E2 antigens in the field of MS.^18^ In that study, on 26 MS cases, 23 healthy controls and 30 patients with collagen disease, significantly more MS patients had a primary antibody response against E2 compared to controls, who had a more pronounced response against E1. All studied E2 epitopes were located within the first 115 AA of the E2 peptide.

Rubella virus is thus a virus of interest in MS, but there is a lack of large‐scale studies assessing its impact. With our previous results on E1 in mind, we aimed to further explore associations between serological responses against the envelope proteins of RV and the risk of developing MS. In this nested case–control study, we utilised a unique material of biobank samples collected before the clinical onset of MS. The material has been extended compared to our previous study on RV in MS.^11^ Here, we aimed to replicate our previous findings on RV E1, and to include serological analysis for anti‐RV E2 antibodies. We further aimed to compare rubella vaccination and infection in this regard. Finally, we aimed to assess associations between serological findings against RV envelope proteins and CNS damage prior to the onset of MS, measured with serum neurofilament light chain (S‐NfL), an established biomarker for axonal damage in the MS prodrome.^19^, ^20^, ^21^

## Results

Cases and controls were well‐matched: mean absolute within‐set difference in sampling date was 4.8 days, and 3.7 months in birth date. Cases were predominantly female (79.5%). Age at sampling, age at onset and time from sampling to onset differed significantly between vaccination cohorts (Table 1). An overview of the seroprevalences against viral agents included in this study is presented in the Supplementary table S3.

**Table 1:** Base data

Cohort	Statistic	Cases	Controls
All	*n*	981	1278
	Median age at sampling, years	22.6 (19.3–26.6)	21.9 (18.8–25.0)
	Median age at disease onset, years	32.1 (27.0–38.3)	n/a
	Median time from sampling until disease onset, years	8.9 (4.0–14.4)	n/a
	Anti‐E1 seroprevalence	88.0%	81.2%
	Median anti‐E1 reactivity in seropositive, MFI	112 (63–215)	96 (55–176)
	Anti‐E2 seroprevalence	47.5%	46.1%
	Median anti‐E2 reactivity in seropositive, MFI	147 (83–283)	141 (83–296)
Unvaccinated	*n*	230	250
	Median age at sampling, years	26.6 (21.0–31.9)	25.9 (20.5–31.3)
	Median age at disease onset, years	39.6 (32.9–44.5)	n/a
	Median time from sampling until disease onset, years	12.4 (5.7–18.8)	n/a
	Anti‐E1 seroprevalence	84.3%	76.1%
	Median anti‐E1 reactivity in seropositive, MFI	166 (74–270)	130 (71–306)
	Anti‐E2 seroprevalence	39.6%	34.3%
	Median anti‐E2 reactivity in seropositive, MFI	129 (82–280)	110 (69–280)
Vaccinated (mono)	*n*	186	208
	Median age at sampling, years	25.0 (22.1–29.5)	24.5 (21.8–29.0)
	Median age at disease onset, years	37.0 (32.4–41.6)	n/a
	Median time from sampling until disease onset, years	9.9 (5.6–14.9)	n/a
	Anti‐E1 seroprevalence	93.5%	89.3%
	Median anti‐E1 reactivity in seropositive, MFI	133 (70–214)	125 (66–205)
	Anti‐E2 seroprevalence	50.0%	49.7%
	Median anti‐E2 reactivity in seropositive, MFI	159 (84–305)	141 (82–314)
Vaccinated (MMR)	*n*	565	820
	Median age at sampling, years	21.1 (18.4–23.9)	20.9 (18.2–23.6)
	Median age at disease onset, years	28.8 (25.5–33.6)	n/a
	Median time from sampling until disease onset, years	7.1 (3.2–12.3)	n/a
	Anti‐E1 seroprevalence	87.6%	80.7%
	Median anti‐E1 reactivity in seropositive, MFI	93 (58–187)	81 (50–150)
	Anti‐E2 seroprevalence	49.9%	49.7%
	Median anti‐E2 reactivity in seropositive, MFI	148 (88–279)	149 (85–297)

Base data for the whole material and stratified for vaccination cohorts. Unvaccinated = presumed to not have received vaccination. Vaccinated (mono) = presumed to have received the monovalent vaccination against rubella virus. Vaccinated (MMR) = presumed to have received the trivalent vaccination against measles, mumps and rubella viruses. Statistics: *n* reported continuously. Age and time variables and seroreactivity reported as median (interquartile range).

E1, rubella virus envelope protein 1; E2, rubella virus envelope protein 2; MFI, median fluorescent intensity; *n*, number of participants; n/a, not available.

Seropositivity for RV E1 was associated with an increased risk of developing MS in both univariate (OR = 1.74, 95% CI 1.35–2.24, *P* < 0.001) and multivariate analysis (adjusted OR [AOR] = 1.67, 95% CI 1.29–2.14, *P* < 0.001; Table 2). This applied not only in the pooled material, but also separately in both the 2012 crosslink and the 2020 crosslink. Vaccination status as co‐factor in the model did not alter the effect size for RV E1. The association between RV E1 and MS risk also remained significant when adjusting for EBNA‐1 seroreactivity quintiles continuously (Supplementary table S4), HLA‐DRB1*15 and HLA‐A*02 carriership, and smoking (Supplementary table S5). In stratified analysis, the association between RV E1 seropositivity and MS risk was significant in both unvaccinated and MMR vaccinated cohorts, but not in the monovalent vaccinated cohort (Table 2). For RV E2, no significant association was found.

**Table 2:** Association between seropositivity for RV E1 or E2 and risk of developing MS

Cohort	Antigen	OR	95% CI	*P*	AOR	95% CI	*P*
All	E1	1.74	1.35–2.24	< 0.001	1.67	1.29–2.14	< 0.001
981 cases, 1278 controls	E2	1.05	0.89–1.24	0.58	1.02	0.86–1.21	0.84
Unvaccinated	E1	1.78	1.08–2.91	0.023	1.78	1.07–2.98	0.028
228 cases, 246 controls	E2	1.23	0.85–1.78	0.27	1.10	0.75–1.63	0.63
Monovalent	E1	1.84	0.86–3.95	0.12	1.52	0.69–3.36	0.30
183 cases, 203 controls	E2	0.99	0.65–1.45	0.95	0.96	0.64–1.45	0.86
MMR	E1	1.73	1.26–2.39	< 0.001	1.71	1.24–2.37	0.001
562 cases, 816 controls	E2	1.02	0.82–1.27	0.88	1.01	0.81–1.27	0.92

Associations between seropositivity for rubella envelope proteins 1 and 2 and the risk of developing MS. Unvaccinated = presumed to not have received vaccination. Vaccinated (mono) = presumed to have received the monovalent vaccination against rubella virus. Vaccinated (MMR) = presumed to have received the trivalent vaccination against measles, mumps and rubella viruses.

AOR, adjusted odds ratio, adjusted for EBV, CMV and HHV‐6A serostatus; CI, confidence interval; E1, rubella virus envelope protein 1; E2, rubella virus envelope protein 2; MFI, median fluorescent intensity; OR, odds ratio.

**Figure 1:**
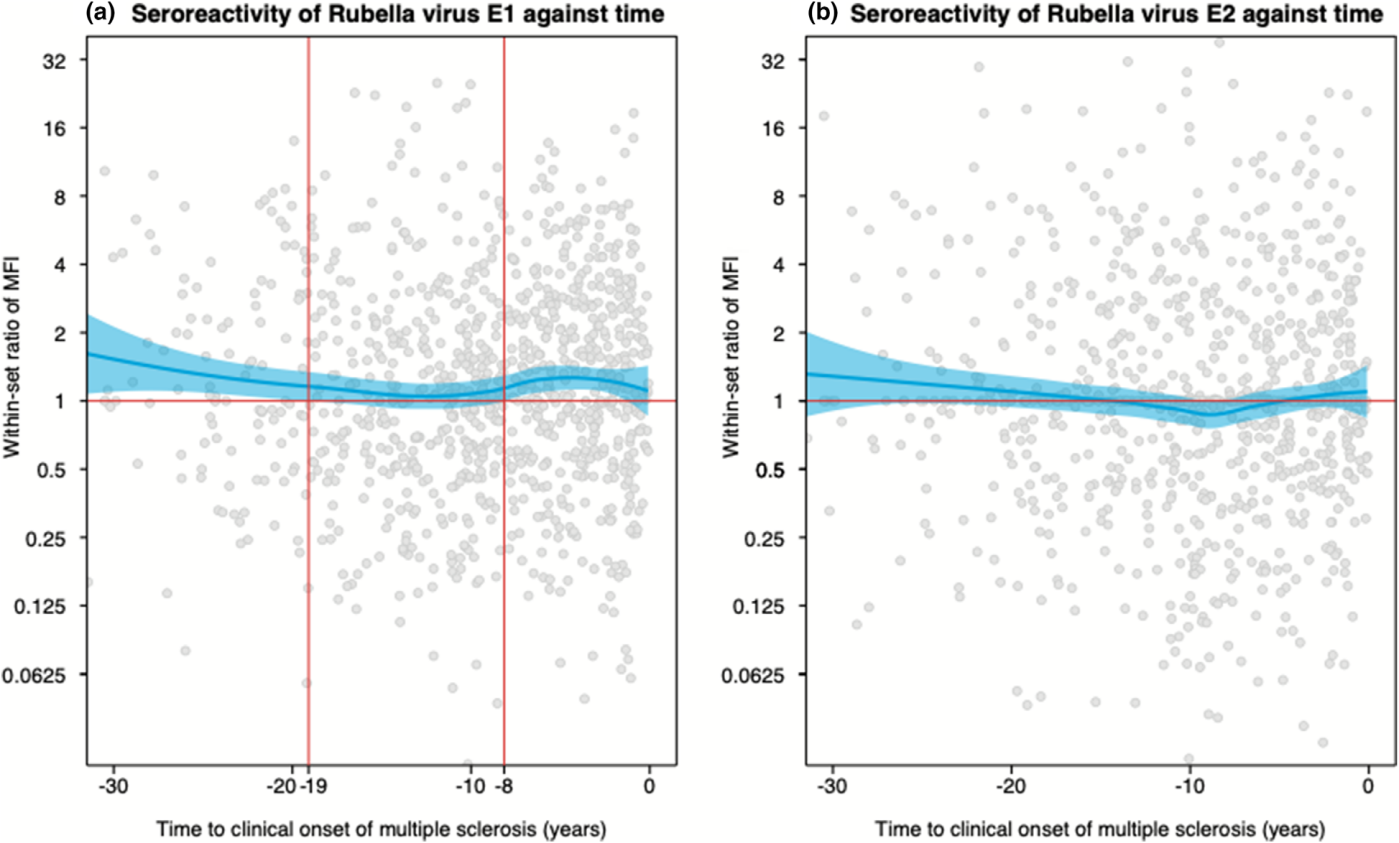
(a and b) Within‐set ratio of seroreactivity against RV E1 and E2 against time to MS onset. Locally estimated scatterplot smoothing plots for within‐set ratio of seroreactivity against rubella virus envelope proteins against time to clinical onset of multiple sclerosis, in the whole population. MFI = median fluorescent intensity, E1 = rubella virus envelope protein 1, E2 = rubella virus envelope protein 2.

Cases seropositive against RV E1 had significantly higher seroreactivity against E1 compared to E1 seropositive controls in unmatched analysis (*P* < 0.001). No such difference in seroreactivity was observed for RV E2, HSV‐1, HSV‐2 or VZV. There was a dose–response relationship between increased RV E1 seroreactivity and the association with MS risk (*P* for trend < 0.001, supplementary table S6). The RV E1 within‐set seroreactivity ratio was significantly increased more than 19 years and again up to 8 years before MS onset (Figure 1). Similar patterns in the last years leading up to MS onset were observed when stratifying for vaccination status (Supplementary figure 3A–C). In samples drawn at 19 years before MS onset or earlier (*n* = 122 cases and 174 matched controls), seroreactivity was significantly higher in cases compared to controls (*P* = 0.045) and seropositivity against E1 was significantly associated with MS risk (OR = 2.9, 95% CI = 1.35–6.23, *P* = 0.006). There was no significant change in E2 ratio at any point in time before MS onset (Figure 1b).

**Figure 2:**
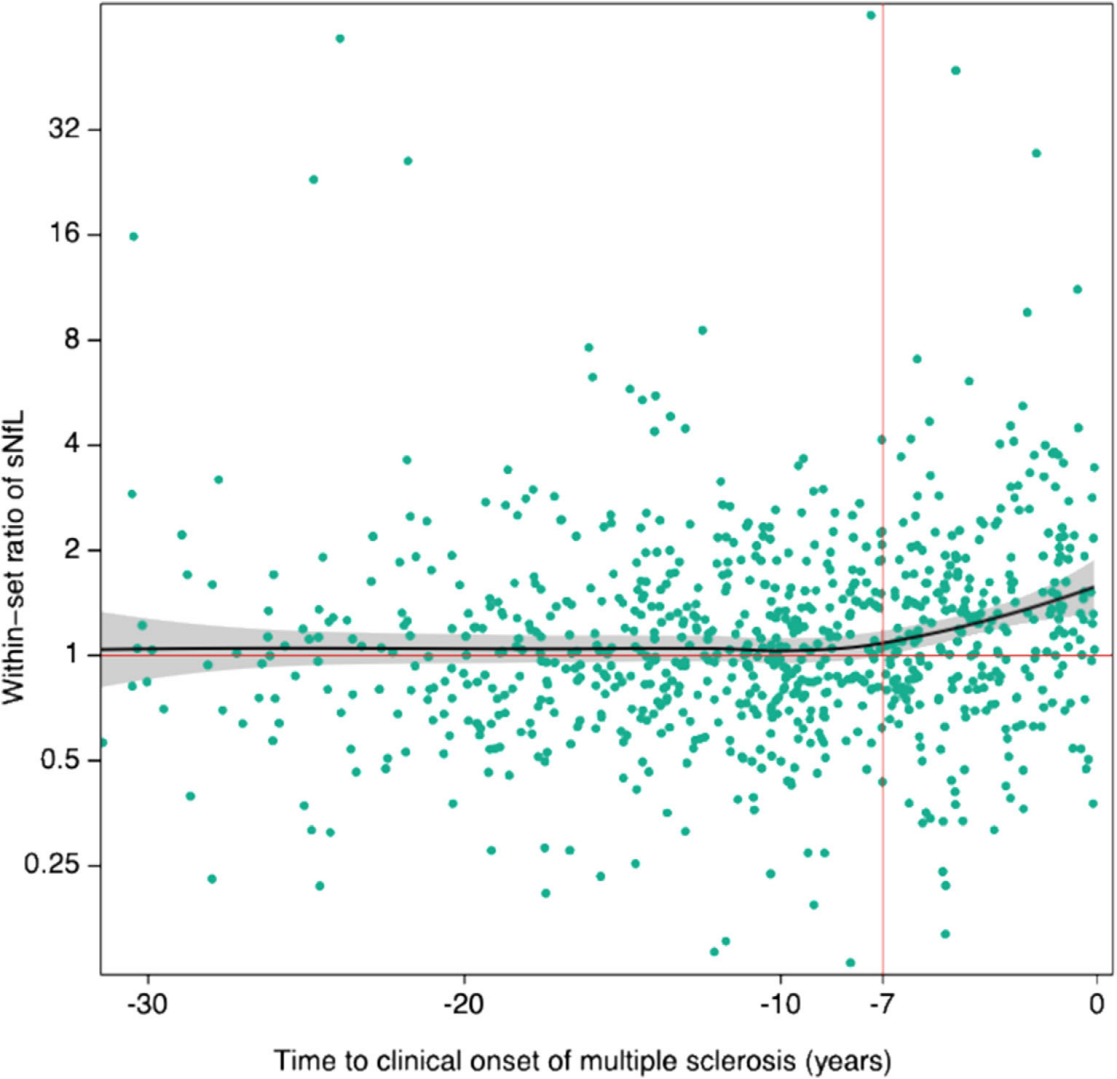
Within‐set ratio of S‐NfL against time to MS onset. Locally estimated scatterplot smoothing plot for within‐set ratio of serum‐neurofilament light chain levels against time before multiple sclerosis onset, in the whole population. S‐NfL = serum‐neurofilament light chain.

The S‐NfL ratio was significantly higher in cases than in controls sampled 7 years or less before the clinical onset of MS (Figure 2). Cases had significantly higher S‐NfL levels compared to controls (6.1 vs 5.5 pg/mL, *P* < 0.001). In cases or controls, no significant associations were observed between RV E1 seropositivity and S‐NfL levels or S‐NfL Z‐score (Table 3), or with the within‐set S‐NfL ratio.

**Table 3:** Rubella virus E1 serostatus and S‐NfL levels in cases and controls

	Cases			Controls		*P*
S–NfL	6.09 (5.83–6.36)			5.45 (5.27–5.64)		< 0.001

	RV E1+	RV E1−	*P*	RV E1+	RV E1−	*P*
S–NfL	6.1 (5.83–6.38)	6.03 (5.18–7.01)	0.86	5.26 (5.08–5.45)	4.99 (4.61–5.40)	0.21
S–NfL Z–score	0.79 (0.69–0.88)	0.66 (0.37–0.96)	0.38	0.47 (0.39–0.55)	0.42 (0.24–0.60)	0.62

Associations between seropositivity for rubella envelope protein 1 and S‐NfL. S‐NfL levels reported as geometric mean (95% confidence interval), measured in pg/mL. S‐NfL ratio reported as geometric mean (95% confidence interval) within‐set ratio between cases and matched controls. S‐NfL Z‐score reported as mean (95% confidence interval). Reported p‐values are two‐sided.

E1−, seronegative for rubella virus envelope protein 1; E1+, seropositive for rubella virus envelope protein 1; S‐NfL, serum‐neurofilament light chain.

## Discussion

In this study on samples collected before the clinical onset of MS, we performed an extended exploration of the association between the immune response to the envelope proteins of RV and the risk of developing MS. We could replicate our previous results,^11^ showing an association between RV E1 seropositivity and the risk of developing MS, both in the new material and the pooled material. The association was robust when adjusting for HHV‐6A, CMV and EBV, as well as for EBNA‐1 seroreactivity, smoking, and HLA‐DRB1*15 and HLA‐A*02 variants. It should be noted that we did not have genetic or smoking data for all participants and therefore could not include these factors in the matched regression analyses because of lack of power. Still, the lack of an effect of HLA variant or smoking on the RV E1 association with MS risk in binary regression argues against their confounding of our results. However, RV E2 was not associated with MS risk. Differences in reactivity levels further supported these findings, as we found that cases had increased reactivity against E1, but not E2, compared to controls.

Furthermore, we divided the material into three cohorts depending on the presumed source of the anti‐rubella antibodies: infection, monovalent rubella vaccination or MMR vaccination. We saw an association between RV E1 seropositivity and MS risk in presumably rubella‐infected subjects and in subjects presumed to have received MMR, but not monovalent rubella vaccination. However, this is explained by differences in power between cohorts.

We could confirm the presence of an increase in S‐NfL levels in the MS prodrome, supporting existing evidence,^20^, ^21^ but found no associations between E1 serostatus and the level of S‐NfL. This lack of an association was probably a consequence of the S‐NfL increase occurring during the 7 years preceding MS onset, while RV seroreactivity first increased many years earlier. Considering these temporal differences, an association between RV serostatus and S‐NfL would not be expected. We saw increased seroreactivity to RV E1 in MS cases both in the 8 years preceding MS, but also in very early samples, 19 years before onset. The pattern in the last years preceding MS onset was similar in the three vaccination cohorts. In other words, we observed a significant increase in rubella E1 seroreactivity before a corresponding increase in S‐NfL, that is preceding axonal damage in the MS prodrome. This temporality argues against RV E1 seropositivity only being an epiphenomenon of a generally increased immune activity in people with MS (pwMS). However, we do not have consecutive samples from the same individuals, and as such, these findings are based on group‐level data. Furthermore, only a smaller subset of samples were drawn at 19 years before MS onset or earlier. Thus, these results should be interpreted with caution.

Our results do not support a role for E2 in the association between RV and MS. However, the E2 assay has several limitations, including low sensitivity, and the antigen only included the first 115 AA of the peptide, although this sequence was specifically chosen based on previous results.^17^, ^18^ Moreover, the E2 assay used in this study was validated against a whole‐virus particle antigen in the reference assay, but *in vivo*, the E2 antigen is obscured.^16^ This may thus explain the low sensitivity in the validation, but also indicates that E2 is not highly immunogenic *in vivo*.^16^, ^22^ A larger concern is that the antigen was expressed in *E. coli*, and therefore, post‐translational modifications such as N‐glycosylation of the recombinant peptide do not reflect E2 expression in human cells. This can affect the peptide folding and thus immunogenicity, especially since RV E2 has several N‐glycosylation sites.^23^ Because of these limitations, our results cannot confidently refute an involvement of RV E2 in the association.

Regarding the E1 assay, false negatives may be common because of the low sensitivity. This raises the concern that if pwMS have a general tendency of increased seroreactivity against any antigen, an assay with low sensitivity will result in higher seroprevalence in cases compared to controls, no matter what antigen was analysed. We have previously argued against this.^11^ If this was the case, one would expect such skewness for E2 in this study, where the sensitivity was low, or for antigens on other viruses such as HSV‐1, HSV‐2 or VZV. Such tendencies were not seen. Importantly, MS cases had increased seroreactivity against E1 compared to controls and a significantly positive mean within‐set ratio of seroreactivity, findings independent of cut‐off.

Hypothetically, there are several potential mechanisms explaining the association between RV E1 and risk of MS. Since E1 has been shown to be able to bind to MOG, oligodendrocyte infection is a possibility.^15^ Such an infection could cause epitope spreading because of potential exposure of self‐antigens after an initial immune response. It could also cause bystander activation, wherein innate immune cells responding to infection, specifically antigen‐presenting cells, can activate CD8^+^ T‐cells, destroying not only the infected cell but also neighbouring non‐infected cells.^24^, ^25^ The RV also has epitopes with homology to several human antigens, although not obviously related to the CNS.^26^ As such, molecular mimicry remains an alternative possibility, also since molecular mimicry may occur even without obvious amino acid sequence homology, but rather because of three‐dimensional similarity.^27^

Controversially, we found an association between RV E1 seropositivity and risk of developing MS in subjects presumed to have received MMR vaccination, which is in line with a recent study on RV E1.^12^ One other study noted that early MMR vaccination was associated with increased risk of MS, but late MMR vaccination was not.^28^ On the contrary, most other studies show no associations between MMR vaccination and risk of MS.^29^ Notably, incidence rates of MS have not drastically changed since the introduction of MMR vaccination, a strong argument against such an adverse effect of the vaccination.^30^, ^31^ Furthermore, several arguments can be made against our results in this particular vaccination cohort. While the method of identifying unvaccinated subjects is reasonably robust, since vaccination was not available when these participants were of an age to be vaccinated, the definition of vaccinated subjects has several limitations thoroughly discussed in our previous paper.^11^ In short, our method presumes vaccination status dependent on birth and sample date. As such, some participants deemed vaccinated in this study may in fact be unvaccinated. However, we have no reason to suspect skewness towards either cases or controls. Moreover, age at vaccination may affect the serological response and, as such, our results. In this respect, the MMR cohort is heterogeneous since it consists of three sub‐cohorts described above. Furthermore, the MMR vaccination contains live, attenuated measles and mumps viruses in addition to RV, introducing factors we cannot adjust for with the current data. Since the rubella vaccine is a live attenuated vaccine, similar serological responses against infection and vaccination would be expected. Although effective, RV vaccination generates a weaker response than infection,^32^, ^33^ and deteriorates over time with individual variation,^32^, ^34^ explaining the observed seroprevalence in the vaccinated cohorts in this study.

One consequence of the Swedish vaccination program is that some individuals in the MMR cohort received their first vaccination at around 12 years of age. For these participants, RV seropositivity may in fact not be the result of only vaccination, as they may have contracted rubella infection prior to vaccination, since RV was still in circulation in Sweden during this time.^35^ Unfortunately, we do not know if this was the case for any given individual. As such, the association in the MMR vaccination cohort may be skewed by pre‐vaccination infection. The same principle can, however, also be applied to the monovalent cohort. Finally, vaccination status did not alter the effect size of RV E1. In other words, the association seen between RV E1 and MS is not dependent on vaccination.

In conclusion, we found an association between MS risk and seropositivity for RV E1 both in presumably infected and vaccinated cohorts. Importantly, our results indicate that people who later develop MS have increased reactivity against RV E1, occurring before evidence of axonal injury in the MS prodrome on group level. Our findings argue against a role of RV E2, although our serological method for E2 has limitations. While EBV remains a crucial viral risk factor for MS, our results support the view that other viruses also may play a role in MS aetiology.

## Method

### Study material

Bio‐banked plasma or serum samples from a total of 1026 individuals who later developed relapsing remitting MS, and 1329 matched controls were included in this Swedish nested case–control study. Participants were born between 1937 and 2000. This material is an extension (‘2020 crosslink’) of a previous material (‘2012 crosslink’), where recruitment and case ascertainment has been described (Supplementary figure 1).^3^, ^10^ Briefly, cases were identified through the Swedish national MS registry and crosslinked to Swedish biobanks. The earliest available biobank sample from the participant, collected before the onset of RRMS and before age 40 years (2012 crosslink) or 25 years (2020 crosslink), was included. Controls were matched on sex, biobank, sampling date and birth date. Cases were matched to 1 control (2012 crosslink) or 1–2 controls (2020 crosslink).

### Vaccination cohorts

The material was divided into cohorts based on vaccination against RV through cross‐checking sampling and birth dates to the Swedish rubella and measles–mumps–rubella (MMR) vaccination programs. Live attenuated rubella vaccination was first offered to girls in 6th grade of school (approximately 12 years) from 1974 to 1982.^35^ Later, it was offered in the trivalent MMR vaccine to all children from 1982 and onwards in a two‐dose regimen.^36^ For the years relevant to this study, one dose was given at 18 months of age, and the second dose in 6th grade of school. Because of the two‐dose regimen, there are three MMR sub‐cohorts (Supplementary table 1), but we combined them into a single MMR cohort next to a monovalent (rubella only) vaccination cohort to retain statistical power. Those with birth or sampling date inconsistent with these cohorts were presumed to be unvaccinated. If a control was not sorted into the same cohort as its matched case, it was excluded from the stratified analysis. If no control of the set remained in the same cohort thereafter, all participants of the set were excluded from the stratified analysis. This removed 21 discordant participants (8 cases and 13 controls).

### Serological analyses and definitions of seropositivity

Serological analyses were performed at the German Cancer Research Center, Heidelberg, using a validated bead‐based multiplex assay.^37^, ^38^ Results were generated for 981 cases and 1278 matched controls. This included serologies for RV E1 and E2, EBV, CMV, HHV‐6A, herpes simplex virus type 1 (HSV‐1), herpes simplex virus type 2 (HSV‐2) and varicella zoster virus (VZV). For all antigens except RV E2 and HHV‐6A, previously defined and validated cut‐offs, measured in median fluorescent intensity (MFI), were used.^37^, ^38^ For HHV‐6A, seropositivity was defined by seroreactivity against the HHV‐6A immediate‐early 1 antigen (IE1A), using the 75th percentile in the controls (MFI 150.25) as cut‐off, as in previous studies.^39^, ^40^, ^41^ Separately, the visual inflection point method^42^ was used to determine HHV‐6A seropositivity, suggesting cut‐off at 150 MFI, supporting the 75th percentile cut‐off.

The serological analysis for RV E2 used the first 115 AA of the E2 peptide as antigen. The assay and validation are described further in the supplementary materials. In brief, the antigen was expressed in *Escherichia coli* and validated against reference sera that had been tested with a whole‐virus antigen assay. To achieve a specificity above 90%, the cut‐off for RV E2 seropositivity was defined as 50 MFI, resulting in a sensitivity of 50.8%. The specificity and sensitivity of the RV E1 assay were 91.7% and 57.6%, respectively.

### Serum neurofilament light chain (S‐NfL)

To assess axonal damage before the onset of MS, samples were analysed for neurofilament light chain (NfL). From a previous study,^7^ levels of S‐NfL were available for 491 cases and 491 controls included in the current study. These were analysed with single molecular array (Simoa™) technology. The same technology was used on the new samples (2020 crosslink), using NF‐light V2 advantage kits analysed on a Simoa HD‐X Analyzer (Quanterix Corporation, Billerica, MA, USA). Sets of cases and controls were analysed on the same plate. In total, S‐NfL levels were available for 843 cases and 1138 controls included in this study. Lack of volume prevented analyses on the remaining samples.

Ratios in S‐NfL were calculated for each individual set of matched cases and controls. For sets with two controls, the geometric mean NfL value for the controls was first calculated to assess this ratio. Furthermore, age‐adjusted S‐NfL Z‐scores were calculated using a reference application.^43^ The application requires BMI, data we did not have, and thus we used a default BMI of 25 for all samples in the Z‐score calculations.

### Epidemiological survey data and HLA data

Data regarding smoking and HLA variants were partially acquired from previous studies.^9^, ^11^, ^44^ Data were complemented using the same standardised questionnaire as in the previous studies, adding results from 31 cases and 135 controls. Combined with the previous material, data on smoking were available for 520 cases and 342 controls. Regarding HLA genotypes, saliva samples were collected from 269 participants, DNA was extracted, and PCR analyses were performed as previously described.^11^ With the addition of these results, data on HLA‐A*02 and HLA‐DRB1*15 genotypes were available for 584 cases, and 307 and 313 controls, respectively.

### Statistics

The associations between RV E1 or E2 serostatus and the risk of developing MS were analysed with conditional logistic regression, calculating odds ratios (ORs) with 95% confidence intervals (CI). The analyses were adjusted for EBV, CMV and HHV‐6A serostatus and stratified for RV vaccination status. Further adjusting for EBNA‐1 seroreactivity quintiles was also performed. The dose–response relationship between RV E1 seroreactivity quintiles and MS risk was also assessed with conditional logistic regression. Quintile cut‐offs were based on the seroreactivity of the controls. Binary logistic regression was performed to adjust for HLA‐A*02 genotype, HLA‐DRB1*15 genotype and smoking.

To calculate seroprevalences adjusted for different numbers of matched controls within a set, the mean serostatus of the controls within a set was first calculated for sets with two controls before calculating seroprevalence. The Kruskal–Wallis *H*‐test was used to compare sample age, time from sampling to onset and age at MS onset between vaccination cohorts. Antibody reactivity against RV E1 and E2, HSV‐1, HSV‐2 and VZV in cases and controls was compared unmatched, with the Mann–Whitney *U*‐test. Mean S‐NfL levels were compared between cases and controls with independent samples *t‐*test in the logarithmic scale. For sets with two controls, the logarithmic mean S‐NfL was first calculated and used in the analysis. Mean S‐NfL levels between rubella E1 seropositive and seronegative individuals were also compared with independent samples *t‐*test in the logarithmic scale. Within‐set seroreactivity ratios were calculated as described for S‐NfL ratios, with the addition that very low reactivity levels were adjusted up to the level of the cut off for each antigen to avoid skewing from extremes. Within‐set ratios of seroreactivity and S‐NfL were plotted against time to the clinical onset of MS and analysed using locally estimated scatterplot smoothing (loess) with 95% CI. One‐sample *t‐*test was used to assess mean within‐set seroreactivity log ratio. All statistical analyses were performed in IBM SPSS statistics version 29.0, except for the loess curves, which were created in R version 4.4.2.

## Conflicts of interest

JI, VG, MB, LPB, TW and SN report no disclosures. PSu serves as an unpaid consultant for Moderna and has received lecture honoraria from Merck. PSt has conducted genetic analysis for Amicus, unrelated to the present work. TO has received advisory board or lecture honoraria from Biogen, Novartis, Merck and Sanofi. The same companies have provided unrestricted MS research grants. IK has received a research grant from Pfizer and a Speaker's honorary from Merck. LA has received lecture honoraria from Biogen, Teva, and Merck.

## Funding

This project was funded by the Swedish Research Council, grant number 2015‐02419. VG was supported by the Research and Development Unit, Region Jämtland Härjedalen, by Oskarfonden, by NEURO Sweden and by The Swedish Foundation for MS Research. PSt was supported by the Deutsche Forschungsgemeinschaft (German Research Foundation) NeuroFlame grant number 523862973, Horizon 2020 MultipleMS grant number 733161, the Swedish Research Council grant number 2020‐01638, and the Neuro Foundation. LA received grants from the Swedish Research Council, grants from the Swedish Research Council for Health Working Life and Welfare, and grants from the Swedish Brain Foundation during the conduct of the study. IK was supported by a Horizon Europe grant for the WISDOM project (grant no 101137154), Swedish Research Council grant no 2020‐01638, and Swedish Brain Foundation.

## Ethics statement

The Regional Ethical Review Board in Umeå approved the project (2011‐198‐31M with subsequent amendments). The study was performed in accordance with the Declaration of Helsinki. All participants were informed via letter and had the possibility to opt out. Study participants who underwent genetic testing signed a written consent form.

## Supporting information


Supplementary figure 1
Supplementary table 1
Supplementary figure 2
Supplementary table 2
Supplementary table 3
Supplementary table 4
Supplementary table 5
Supplementary table 6
Supplementary figure 3

## Data Availability

The data that support the findings of this study are available on request from the corresponding author. The data are not publicly available due to privacy or ethical restrictions.
