# Supplementary material for: Seropositivity against rubella virus envelope protein 1, but not 2, is associated with an increased risk of multiple sclerosis
Source: Clin Transl Immunology. 2026 Apr 16;15(4):e70094. doi: 10.1002/cti2.70094 (PMC13084256; doi:10.1002/cti2.70094)
Supplement: Supplementary file 1 — Supplementary figure 1 Supplementary table 1 Supplementary figure 2 Supplementary table 2 Supplementary table 3 Supplementary table 4 Supplementary table 5 Supplementary table 6 Supplementary figure 3 [file CTI2-15-e70094-s001.docx]

**Supplementary materials:** Seropositivity for rubella virus envelope protein 1, but not 2, is associated with an increased risk of multiple sclerosis

**Contents:**

- **Supplementary figure S1: Case ascertainment flow chart.**
- **Supplementary table S1: Measles-mumps-rubella vaccination sub-cohorts.**
- **Validation experiment for rubella virus envelope protein 2**
- **Supplementary figure S2: Antibody response [MFI] to Rubella protein E2 stratified by reference assay serostatus. The dashed line indicates the cut-off defining at least 90% specificity.**
- **Supplementary table S2:** **Contingency table and summary statistics showing concordantly and disconcordantly reacting sera in Rubella multiplex serology in comparison to reference assay serostatus.**
- **Supplementary table S3: seroprevalences against viral agents in cases and controls.**
- **Supplementary table S4: Conditional logistic regression calculating associations between seropositivity for RV E1 and the risk of developing MS, adjusted for CMV and HHV-6A seropositivity and EBNA-1 reactivity quintiles.**
- **Supplementary table S5: Binary logistic regression calculating associations between seropositivity for RV E1 and the risk of developing MS, adjusted for HLA-A*02, HLA-DRB1*15 and smoking as well as EBV, CMV and HHV-6A.**
- **Supplementary table S6: Conditional logistic regression relating quintile of seroreactivity against rubella virus E1 and MS risk.**
- **Supplementary figure 3A-C: Within-set-ratio for seroreactivity against RV E1 over time in the three vaccination cohorts.**

**Supplementary figure S1:** **Case ascertainment flow chart.**
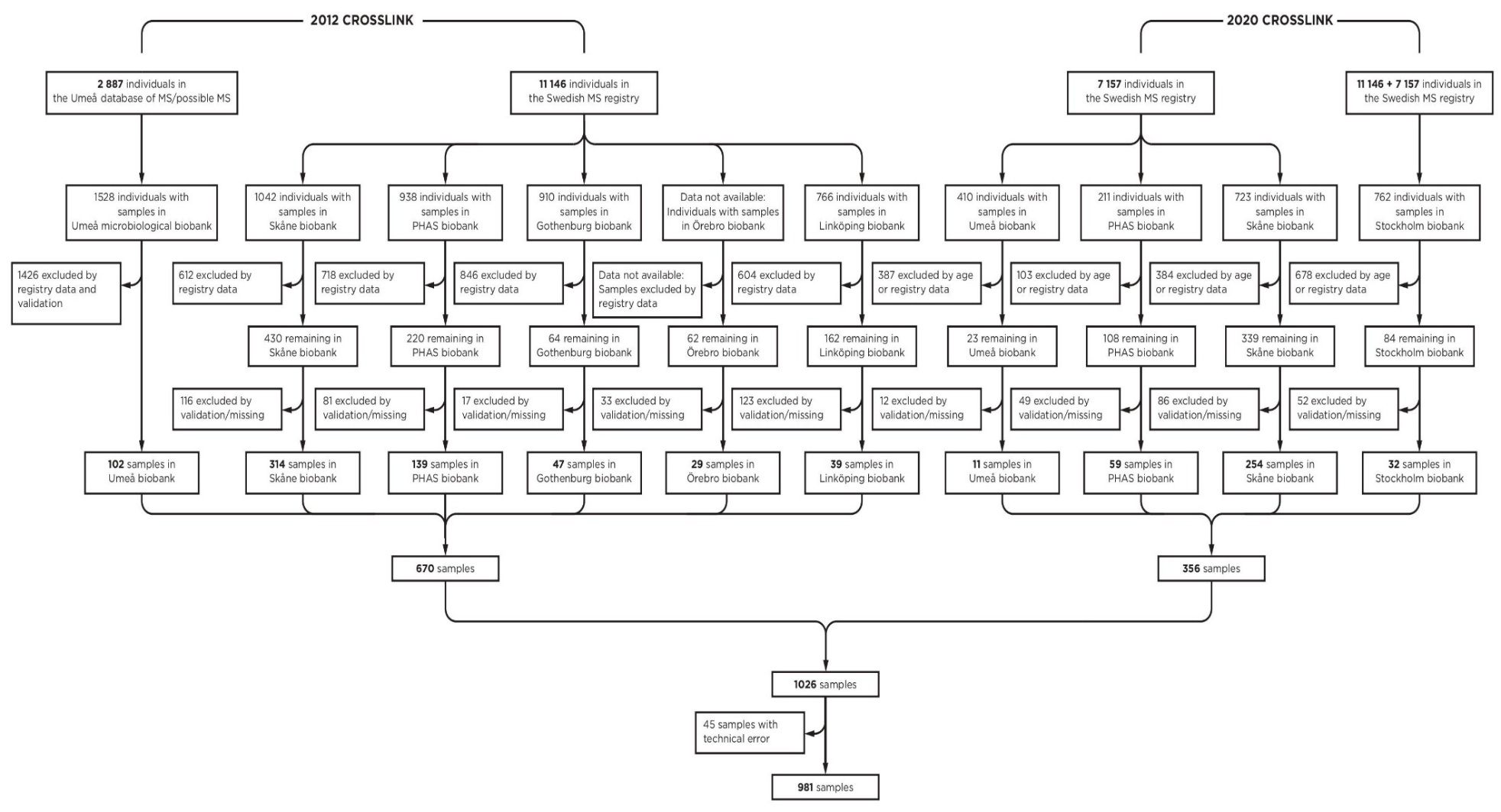


**Note:** Flow chart for case ascertainment in the two materials (previous material of 670 cases vs extension material of 356 cases), and their pooling for the current study. **Abbreviations:** MS = multiple sclerosis, FHM = Swedish public health agency.

**Supplementary table S1: Measles-mumps-rubella vaccination sub-cohorts.**

| **Cohort** | **Cases (n)** | **Controls (n)** |
| --- | --- | --- |
| MMR late | 312 | 382 |
| MMR early | 18 | 23 |
| MMR double | 235 | 396 |

**Note:** Due to introducing the measles-mumps-rubella (MMR) vaccination in a two-dose regime, at both 18 months and in grade 6 of school (approximately 12 years old), there are three MMR sub-cohorts. A “standard” sub-cohort, consisting of those vaccinated at 18 months and in 6^th^ grade (“MMR double”). A second sub-cohort, consisting of those aged above 18 months but not yet in 6^th^ grade when the vaccination was introduced, who only received a single MMR dose in 6^th^ grade (“MMR late”). Finally, a third sub-cohort with participants sampled after 18 months of age but before they reached 6^th^ grade, who had only received the first dose of MMR vaccine when the sample included in this study was collected (“MMR early”). We did, however, not have enough participants to divide the material into these three sub-cohorts without losing too much power and introduce further multiple testing issues and therefore combined them into a single MMR cohort. Samples of sets not sorted into the same cohort are excluded. **Abbreviations:** MMR = measles-mumps-rubella (vaccination).

**Validation experiment for rubella virus envelope protein 2**

The selected amino acid sequence for E2 (Uniprot accession # P08563, strain M33, amino acids 301-415 in polyprotein) was recombinantly expressed as Glutathione-S-transferase (GST)-tagged protein in *E. coli* BL21 and applied in multiplex serology as described elsewhere.^1^

The reference serum sample set was the same as applied in Brenner et al.^2^ with n=33 reference ELISA tested negative individuals and n=65 reference ELISA (Enzygnost® Anti-Rubella-Virus/IgG immunoassay, Siemens Healthcare Diagnostics Products GmbH, Marburg, Germany) tested positive individuals. Reference samples were tested at serum dilution 1:1000 for antibodies to Rubella protein E2. Testing and first data evaluation took place blinded for reference assay serostatus. For further statistical analysis, reference assay serostatus was unblinded. At a cut-off for sero-positivity that defines at least 90% specificity (Figure S2), the sensitivity to E2 in 1:1000 serum dilution was 50.8% (table S1).

**Supplementary figure S2:** **Antibody response [MFI] to Rubella protein E2 stratified by reference assay serostatus. The dashed line indicates the cut-off defining at least 90% specificity.**


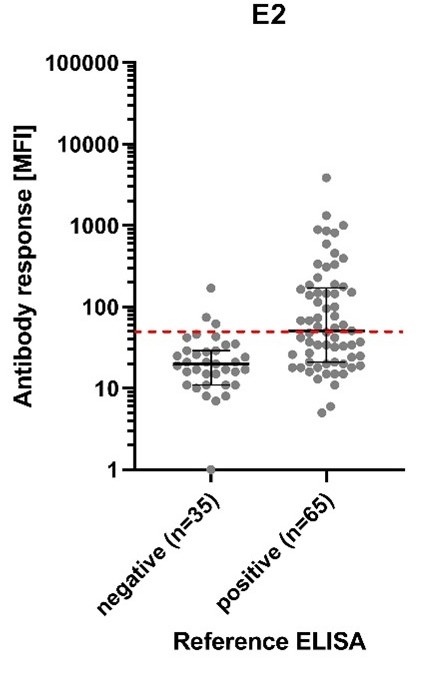


**Supplementary table S2:** **Contingency table and summary statistics showing concordantly and discordantly reacting sera in Rubella multiplex serology in comparison to reference assay serostatus.**

|  | Cut-off | Ref ELISA neg (n=33) | Ref ELISA pos (n=65) | Sensitivity (%) | Specificity (%) | Agreement  *Kappa* (95% CI) |
| --- | --- | --- | --- | --- | --- | --- |
| E2 + | 50 | 3 | 33 | 50.8 | 91.4 | 0.35 (0.21, 0.50) |

**Supplementary Table S3: Seroprevalences against viral agents in cases and controls.**

| **Agent** | **Cases** | **Controls** |
| --- | --- | --- |
| RV E1 | 88.0% | 81.2% |
| RV E2 | 47.5% | 46.1% |
| EBV | 91.7% | 87.6% |
| HHV-6A | 34.3% | 25.8% |
| CMV | 50.1% | 58.3% |
| VZV | 73.6% | 75.2% |
| HSV-1 | 89.6% | 88.4% |
| HSV-2 | 14.1% | 13.4% |

**Note:** Seroprevalences against the analysed antigens on rubella virus and other viral agents included in this study. **Abbreviations:** RV E1 = rubella virus envelope protein 1, RV E2 = rubella virus envelope protein 2, EBV = Epstein-Barr virus, HHV-6A = human herpesvirus 6A, CMV = cytomegalovirus, VZV = varicella zoster virus, HSV1 = herpes simplex virus type 1, HSV2 = herpes simplex virus type 2.

**Supplementary table S4: Conditional logistic regression calculating associations between seropositivity for RV E1 and the risk of developing MS, adjusted for CMV and HHV-6A seropositivity and EBNA-1 reactivity quintiles (n=981 cases and 1278 controls).**

|  |  | **OR** | **p** | **95% CI** |
| --- | --- | --- | --- | --- |
| **RV E1 +** |  | 1.56 | <0.001 | 1.20–2.04 |
| **HHV-6A +** |  | 1.36 | 0.004 | 1.10–1.67 |
| **CMV +** |  | 0.64 | <0.001 | 0.53–0.77 |
| **Anti-EBNA 1 reactivity (quintiles)** |  | 1.42 | <0.001 | 1.33–1.52 |
|  |  |  |  |  |

**Note:** Conditional logistic regression calculating associations between seropositivity for RV E1 and the risk of developing MS, adjusted for CMV and HHV-6A seropositivity and EBNA-1 reactivity quintiles. EBNA-1 reactivity quintiles based on seroreactivity in controls. **Abbreviations:** RV = rubella virus, E1 = envelope protein 1, HHV-6A = human herpesvirus 6A, CMV = cytomegalovirus, EBNA-1 = Epstein Barr nuclear antigen 1, OR = odds ratio, CI = confidence interval, MFI = median fluorescense intensity.

**Supplementary table S5: Binary logistic regression calculating associations between seropositivity for RV E1 and the risk of developing MS, adjusted for HLA-A*02, HLA-DRB1*15 and smoking as well as EBV, CMV and HHV-6A (n=468 cases and 281 controls).**

|  | OR | p | 95% CI |
| --- | --- | --- | --- |
| RV E1 + | 1.62 | 0.037 | 1.03–2.54 |
| HLA-A*02 + | 0.50 | <0.001 | 0.36–0.70 |
| HLA-DRB1*15 + | 3.12 | <0.001 | 2.26–4.32 |
| Smoking (ever) | 1.92 | <0.001 | 1.39–2.65 |

**Note:** Binary logistic regression calculating associations between seropositivity for rubella envelope protein 1, carriership (homo or heterozygote) for HLA-A*02 or HLA-DRB1*15, and smoking (ever smoker) and the risk of developing MS. Odds ratios also adjusted for serostatus against Epstein-Barr virus (EBV), cytomegalovirus (CMV) and human herpesvirus 6A (HHV-6A). Only individuals with data available for all factors are included. **Abbreviations:** RV = rubella virus, E1 = envelope protein 1, HLA = human leukocyte antigen, OR = odds ratio, CI = confidence interval.

**Supplementary table S6: Conditional logistic regression relating quintile of seroreactivity against rubella virus E1 and MS risk.**

|  |  | **Quintiles of RV E1 seroreactivity** | |  |  |  |
| --- | --- | --- | --- | --- | --- | --- |
|  | **p for trend** | **1** | **2** | **3** | **4** | **5** |
| Univariate | <0.001 | Reference | 1.38 (1.01–1.87) | 1.88 (1.39–2.53) | 1.66 (1.22–2.24) | 2.14 (1.58–2.90) |
| Multivariate* | <0.001 | Reference | 1.32 (0.95–1.82) | 1.62 (1.19–2.22) | 1.45 (1.06–2.01) | 1.92 (1.39–2.65) |

**Note:** Conditional logistic regression relating quintile of seroreactivity against rubella virus E1 and MS risk, reporting odds ratio with 95% confidence interval in for each quintile compared to the lowest, reference quintile. *Adjusted for serostatus against cytomegalovirus and human herpesvirus 6A, and continuously for quintiles of seroreactivity against Epstein-Barr virus nuclear antigen 1. **Abbreviations:** RV E1 = rubella virus envelope protein 1.

**Supplementary figure 3A-C:** **Within-set-ratio for seroreactivity against RV E1 over time in the three vaccination cohorts.**

**Note:** Locally estimated scatterplot smoothing plots for geometric mean within-set-ratio of seroreactivity against rubella virus envelope protein 1 against time to clinical onset of multiple sclerosis in the three vaccination cohorts. **A:** Unvaccinated = presumed to not have received vaccination. **B:** Monovalent vaccine = presumed to have received the monovalent vaccination against rubella virus. **C:** MMR vaccine = presumed to have received the trivalent vaccination against measles, mumps and rubella viruses. **Abbreviations:** MFI = median fluorescent intensity, RV E1 = rubella virus envelope protein 1, MMR = measles, mumps and rubella vaccination.

**References**

1. Waterboer T, Sehr P, Michael KM, et al. Multiplex human papillomavirus serology based on in situ-purified glutathione s-transferase fusion proteins. *Clin Chem*. 2005;51(10):1845-1853. doi:10.1373/CLINCHEM.2005.052381

2. Brenner N, Butt J, Bomfim IL, et al. Validation of monoplex assays detecting antibodies against Corynebacterium diphtheriae and Clostridium tetani toxins, rubella virus and parvovirus B19 for incorporation into Multiplex Serology. *Methods*. 2019;158:44-53. doi:10.1016/j.ymeth.2019.01.013
